# A Rare Cause of Precocious Puberty: Hepatoblastoma

**DOI:** 10.4274/jcrpe.v1i6.281

**Published:** 2010-12-08

**Authors:** Erdal Eren, Metin Demirkaya, Esra D. Papatya Çakır, Betül Sevinir, Halil Sağlam, Ömer Tarım

**Affiliations:** 1 Department of Pediatric Endocrinology, Uludağ University, Faculty of Medicine, Bursa, Turkey; 2 Department of Pediatric Oncology, Uludağ University, Faculty of Medicine, Bursa, Turkey; +90 224 295 05 40+90 224 442 81 43erderen@yahoo.comUludağ University, Medical Faculty, Department of Pediatric Endocrinology, Bursa, Turkey

**Keywords:** puberty, hepatoblastoma

## Abstract

Hepatoblastoma, an embryonal tumor, is one of the most common primary liver tumors in childhood. It secretes human chorionic gonadotropin (hCG), which can cause precocious puberty (PP). Herein, we present a case with PP who had enlarged penile size noticed during a diagnosis of hepatoblastoma. Laboratory examination revealed increased testosterone, alpha-fetoprotein (AFP), and hCG levels. Serum follicle-stimulating hormone (FSH) and luteinizing hormone (LH) levels were within prepubertal ranges. The diagnosis of hepatoblastoma was made by liver biopsy. Chemotherapy was administered, and the patient was referred to surgery. Ten months later, testis volumes were below 4 ml bilaterally, and penile length was 5.5 cm. Serum testosterone, AFP, and hCG levels decreased. Resection of the tumor and chemotherapy are essential for the treatment of hepatoblastoma and they can eliminate the symptoms of PP.

**Conflict of interest:**None declared.

## INTRODUCTION

Precocious puberty (PP) is a term that defines the onset of signs of puberty before age 8 in girls and age 9 in boys. Sex steroid production in these children can stem from hypothalamic-pituitary-gonadal axis activation (GnRH-dependent PP), or may be due to a nonhypothalamic-mediated increase in sex steroid production (GnRH-independent PP). In patients presenting with precocious puberty, more than 90% of girls and about half of boys have GnRH-dependent PP (1).

Hepatoblastoma, embryonal tumor, is one of the most common primary liver tumors in childhood. It secretes human chorionic gonadotropin (hCG), which can cause GnRH-independent PP. It is more common in males. Mean age at presentation of this liver tumor, occurring predominantly in childhood, is 18 months. It may be seen in newborn babies which suggests that its origin may be prenatal (2, 3). So far, over twenty cases with PP associated with hepatoblastoma have been reported. The aim of this presentation was to revisit a known, but rare cause of PP.

## CASE REPORT

**Figure 1:**
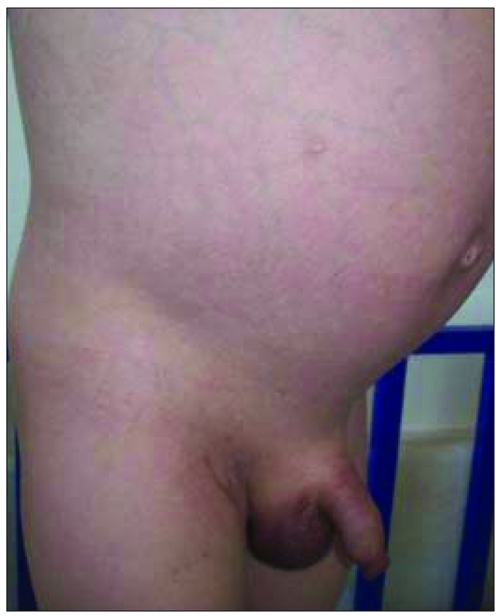
Marked abdominal distention and visible peripheral veins

A three-year-old boy was presented to the hospital because of abdominal distention, hair on the scrotal area, and enlarged penile size, noticed 2 months prior to the presentation. Ultrasonography demonstrated a liver mass, and the patient was referred to the Department of Pediatric Oncology. Protuberant abdomen, hepatomegaly (palpable 14 cm below the costal margin), scrotal hair, enlarged testes (volume 6 ml/6 ml), and enlarged penile size (8 cm) were noted on physical examination (Figure 1 and 2). Laboratory investigation revealed a leukocyte count of 14 100/mm^3^, a platelet count of 83 500/mm^3^, normal transaminase levels, increased testosterone (4.02 ng/ml; normal <0.1 ng/ml), alpha-fetoprotein (AFP) (>30 000 ng/ml; normal 3-14 ng/ml) and hCG (33.1 IU/L; normal 0-5 IU/L) levels. Serum follicle-stimulating hormone (FSH) and luteinizing hormone (LH) levels were in the prepubertal ranges. Ultrasonographic examination showed a heterogenic liver tissue and abdominal free fluid. The diagnosis of hepatoblastoma was made by liver biopsy. Chemotherapy (the PLADO protocol) was administered, and the patient was referred to surgery. Ten months later, testis volumes were below 4 ml bilaterally, and penile length was 5.5 cm. Serum testosterone, AFP, and hCG levels decreased (0.14 ng/mL, 1.2 ng/mL, 5.41 IU/L, respectively) to normal ranges.

**2:**
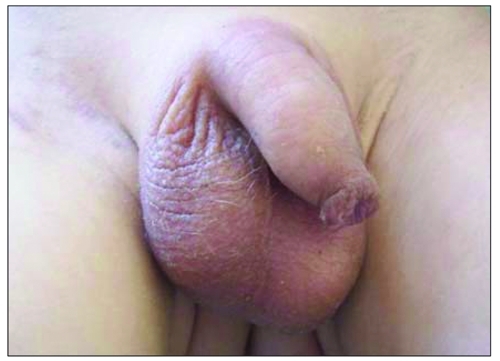
Increased testicular volume, penile length, and scrotal hair

## DISCUSSION

Liver tumors account for one percent of all malignancies in childhood, and 1.4 in one billion children under 16 years is re ported to be diagnosed with liver tumor every year. The incidence of hepatoblastoma is increased by 15 fold in babies with birth weights less than 1000 grams. The tumor is rare after the age of 3 years.

Liver tumor risk increases in some diseases, such as Beckwith-Wiedemann syndrome, familial adenomatous polyposis, trisomy 18, glycogen storage diseases, hereditary tyrosinemia type 1, and Alagille syndrome increase the risk of liver tumor. Most cases are admitted to hospital because of abdominal mass, abdominal pain, weight loss, anorexia, nausea and vomiting. Serum AFP and hCG levels are increased in most patients and  are important both for diagnosis and follow up. The-five year survival rate of patients with hepatoblastoma is between 65-75% after resection of the tumor and chemotherapy (3).  

The first case with PP due to hepatoblastoma was reported in 1963 (4). Approximately 20 cases have been reported so far. Heimann et al. (5) have reported a 2-year-old boy with PP whose AFP, and hCG levels had decreased after liver Tx. Similarly, another patient reported by Heinrich et al (6) had decreased AFP levels after tumor resection and chemotherapy. Tumor markers and testosterone levels also decreased in our patient after chemotherapy. An African boy with isosexual PP associated with hCG-secreting hepatoblastoma was presented in 2008 (7).  Hepatoblastoma can secrete hCG, which is biologically similar to LH and stimulates Leydig cells in the testes. Furthermore, it was shown that hepatoblastoma may also secrete testosterone. Galifer (8) has presented a patient with PP and testosterone-secreting hepatoblastoma.

PP due to liver tumor in childhood is not a frequently encountered condition. Giacomantonio (9) presented only 3 patients with PP among 48 cases of primary liver tumor. 

In conclusion, we want once again to draw attention to hepatoblastoma, which should be kept in mind as a cause of PP by the clinicians in gastroenterology, oncology, and endocrinology.
